# Addressing diagnostic challenges in neglected Bosworth dislocation with open reduction and soft tissue reconstruction: a case report

**DOI:** 10.1097/RC9.0000000000000187

**Published:** 2026-02-16

**Authors:** Hilmi Muhammad, Andi Muhammad Mukhsin Putrama, A Faiz Huwaidi

**Affiliations:** a Pediatric Sub-Division, Orthopaedics and Traumatology Division, Department of Surgery, Faculty of Medicine, Public Health and Nursing, Universitas Gadjah Mada/Dr. Sardjito General Hospital, Yogyakarta, Indonesia; b Resident of Orthopaedics and Traumatology Division, Department of Surgery, Faculty of Medicine, Public Health and Nursing, Universitas Gadjah Mada/Dr. Sardjito General Hospital, Yogyakarta, Indonesia

**Keywords:** Bosworth fracture-dislocation, rare case, surgical technique

## Abstract

**Introduction and importance:**

Bosworth fracture-dislocation is a rare and complex ankle injury characterized by the distal fibular fragment becoming entrapped behind the tibia, often following an oblique fibular fracture. Due to its unique anatomical presentation, the injury is frequently misdiagnosed or overlooked, particularly in chronic or neglected cases, leading to long-term complications. This case report explores a novel surgical technique for managing chronic Bosworth fracture-dislocation.

**Case presentation:**

An 18-year-old woman presented with persistent pain and difficulty bearing weight on her left ankle 6 months after a motor vehicle accident. Initial evaluations missed the diagnosis, but X-rays revealed a posterior dislocation of the distal fibula behind the tibia, confirming Bosworth fracture-dislocation. Given the chronicity of the injury, surgical intervention was required. The patient underwent a direct lateral approach to reduce the dislocated fibula, followed by fixation with two 4.0-mm syndesmotic screws and augmentation using a split peroneus brevis tendon graft for enhanced lateral ankle stability.

**Clinical discussion:**

Bosworth fracture-dislocations are challenging to manage due to the difficulty of reducing the fibula and the lack of consensus on the optimal surgical approach. The failure of closed reduction techniques necessitates open reduction and internal fixation. This case utilized a combined approach of syndesmotic screw fixation and soft tissue reconstruction. This technique effectively restored both bony alignment and soft tissue integrity.

**Conclusion:**

This case report highlights the diagnostic difficulties and rarity of Bosworth fracture-dislocation, especially in chronic cases. This surgical technique offers a successful approach for managing chronic Bosworth fracture-dislocation.

## Introduction

Bosworth fracture-dislocation is a rare and complex ankle injury where the distal fibular fragment becomes entrapped behind the tibia following an oblique fibular fracture. This occurs when the proximal fibular fragment is displaced and wedged behind the posterolateral ridge of the lateral tibial tubercle, creating a mechanical obstruction that makes conventional closed reduction techniques ineffective, requiring open surgical intervention for definitive management. In a cohort of over 3000 ankle fracture patients, approximately 1.62% were diagnosed with Bosworth fracture-dislocation, suggesting it may be more common than traditionally recognized. However, the rarity of the injury and its unique anatomical presentation make diagnosis challenging, often leading to delayed or missed diagnoses. Inadequate initial management can cause the injury to progress into a chronic or neglected state, significantly complicating treatment and recovery. Failure to promptly diagnose and treat Bosworth fracture-dislocation, particularly in emergency settings, may result in long-term complications such as joint instability, persistent pain, and permanent functional impairment^[1,2,3]^.

**HIGHLIGHTS**

- Bosworth dislocation is a rare ankle injury where the distal fibula becomes entrapped behind the tibia, often misdiagnosed, particularly in chronic cases.
- A surgical technique involving open reduction, syndesmotic screw fixation, and peroneus brevis tendon reconstruction successfully treated chronic Bosworth dislocation in a young female patient.
- This case highlights the diagnostic challenges and the lack of standardized treatment, offering a new approach for managing chronic Bosworth dislocation with favorable outcomes.

For chronic or neglected cases of Bosworth fracture-dislocation, the current literature lacks a clear consensus on the optimal surgical approach and the necessary preoperative planning for successful outcomes. The absence of standardized treatment guidelines highlights the complexity of managing such cases and underscores the need for individualized surgical decision-making, based on intraoperative findings and anatomical distortions^[4]^. This study reports the successful surgical management of a Bosworth fracture-dislocation, contributing valuable insight into the management of this rare injury. This case report has been reported in line with the SCARE Checklist^[5]^.

## Case presentation

An 18-year-old woman presented to the outpatient clinic with persistent discomfort and difficulty weight-bearing on her left ankle, which had progressively affected her mobility. The patient reported that these symptoms began 6 months ago following a motor vehicle accident. During the initial evaluation after the accident, the patient was informed that no fractures, dislocations, or significant injuries were detected in her extremities.

**Figure 1.**
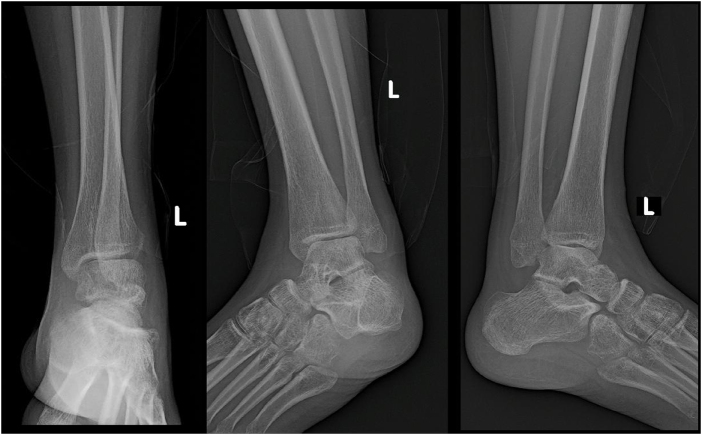
Preoperative X-ray: This image shows a posterior dislocation of the distal fibula behind the tibia, with no visible fracture lines..

Due to the persistent symptoms and lack of improvement, the patient sought further medical assessment at our center. An X-ray of the ankle revealed a posterior dislocation of the distal fibula behind the tibia, without any visible fracture lines (Fig. 1). Based on this finding, we diagnosed the patient with Bosworth fracture-dislocation. Given the chronic and neglected nature of the injury, surgical intervention was deemed necessary to restore ankle stability and function.

**Figure 2.**
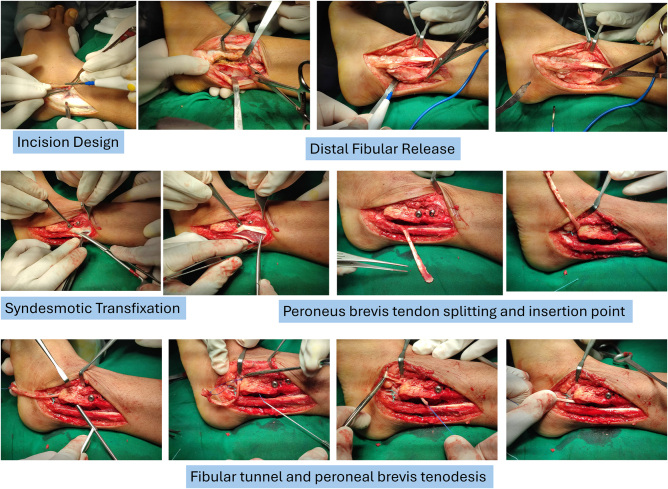
Surgical technique: We performed a distal fibular release, syndesmotic transfixation, and augmentation using the peroneus brevis tendon.

A direct lateral approach to the distal fibula was performed to provide optimal exposure of the affected anatomical structures. During the procedure, extensive fibrous tissue surrounding the dislocated fibular fragment was identified, as we had anticipated due to the neglected condition. We assessed the tendons, ligaments, and neurovascular structures to confirm the diagnosis and evaluate the preoperative condition. We carefully released and debrided the fibrous tissue to facilitate mobilization. After thorough soft tissue clearance, the distal fibula was anatomically reduced and confirmed under fluoroscopic guidance to restore normal ankle mortise alignment. Rigid fixation was achieved using two 4.0-mm cannulated syndesmotic screws to stabilize the distal tibiofibular joint and promote ligamentous healing. To further enhance lateral ankle stability, a tenodesis procedure was performed using a split peroneus brevis tendon graft. The tendon was harvested and transposed to augment the distal fibula, reinforcing the lateral ligamentous complex (Fig. 2). This combined approach aimed to restore both bony congruity and soft tissue integrity, optimizing functional recovery.

The peroneus brevis tendon was selected for augmentation because it provides a strong, locally available autograft with low donor-site morbidity and good biomechanical compatibility for ankle ligament reconstruction. Its effectiveness has been demonstrated with mechanical stability at follow-up^[6]^. The anatomic reconstruction using a split peroneus brevis graft restored ankle stability while preserving subtalar motion^[7]^. In addition to supporting these established findings, the present case contributes new clinical insight by demonstrating that the peroneus brevis tendon can be effectively used as an augmentation option in the setting of a neglected Bosworth fracture-dislocation.

**Figure 3.**
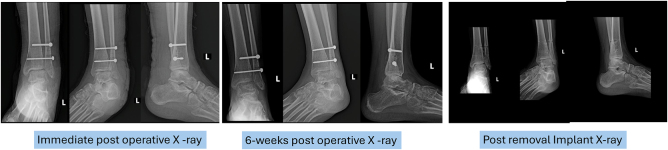
Postoperative X-ray: The immediate postoperative X-ray, the X-ray taken 6 weeks postoperatively, and the X-ray taken after implant removal all show satisfactory anatomical alignment..

**Figure 4.**
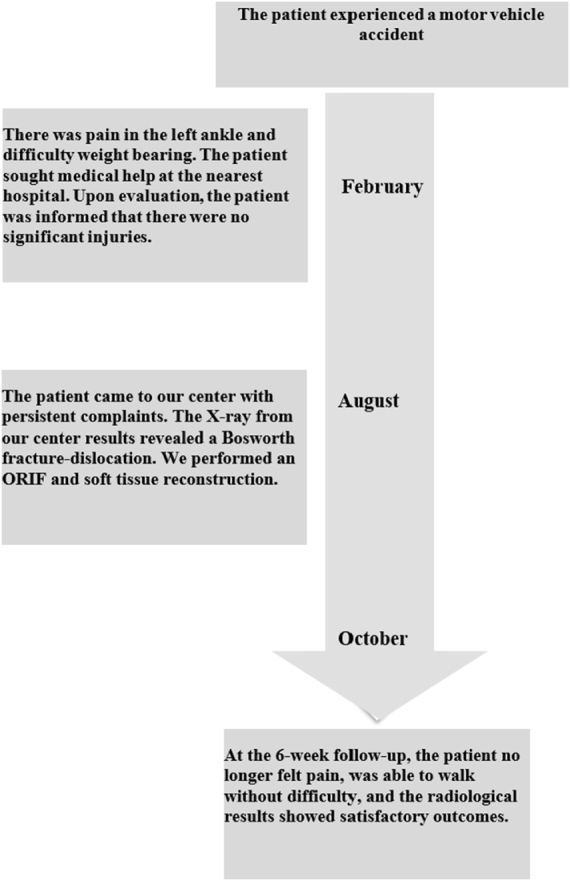
The timeline of the patient’s clinical progression.

Postoperative radiographs confirmed satisfactory anatomical alignment of the distal tibiofibular joint (Fig. 3). The two cannulated syndesmotic screws were appropriately positioned and maintained stable fixation, with no evidence of hardware loosening or malposition. At the 1-month follow-up, the patient reported complete resolution of pain and discomfort, with restored ambulatory function and no signs of instability or mechanical limitation. By 6 weeks postoperatively, clinical examination revealed the absence of tenderness or swelling around the ankle. The patient demonstrated full, pain-free range of motion, indicating a successful early functional recovery. The timeline of the patient’s clinical progression is depicted in Fig. 4.

This surgical technique, combining open reduction, syndesmotic screw fixation, and soft tissue reconstruction using the peroneus brevis tendon, presents a novel approach in the management of chronic and neglected Bosworth fracture-dislocation, offering significant improvements in both bony stability and soft tissue function.

## Discussion

The management of Bosworth fracture-dislocations has generated considerable debate among practitioners, largely due to the technical challenges encountered during reduction. Closed reduction techniques are frequently ineffective, with a documented failure rate exceeding 70%. This is typically attributed to the entrapment of the fibula and the surrounding soft tissues interposed between the bones^[8,9]^. Consequently, open reduction and internal fixation (ORIF) is often recommended as the preferred treatment when closed reduction fails. Such surgical interventions are crucial not only for restoring anatomical alignment but also for reducing the risk of complications such as compartment syndrome, which can arise from prolonged dislocation^[10,11]^.

Surgical management of chronic or neglected Bosworth fracture-dislocations presents unique challenges due to the long-standing nature of the injury, potential soft tissue complications, and anatomical deformities that develop from the prolonged dislocation. The chronicity of Bosworth fracture-dislocations, marked by the fibular entrapment behind the tibia, necessitates a nuanced surgical approach to effectively restore anatomical integrity and function.

The primary surgical approach involves ORIF of the fibula, which is crucial to overcome the resistance caused by the intact interosseous membrane and the surrounding soft tissues^[12]^. A meticulous surgical technique is necessary for effective reduction and fixation. Once the incision is made, the posterior capsule must be carefully assessed and any interposed soft tissue meticulously removed to facilitate the reduction of the fibula and restore normal anatomic alignment^[13,14]^. In this case, we use two syndesmotic screws to maintain the reduction and augment it with peroneal brevis tendon. This is the surgical technique not commonly used for managed Bossworth dislocation and in our experience gives the satisfactory outcomes.

Management of chronic or neglected Bosworth fracture-dislocations is challenging, and published surgical strategies vary depending on the degree of fibular entrapment and soft-tissue compromise. ORIF alone remains effective in acute cases, with good union and functional outcomes achieved when early reduction is possible^[6]^; however, neglected cases frequently require more extensive approaches such as a posterolateral/posterior exposure to disengage the fibula or fibular osteotomy when dense fibrosis prevents mobilization^[6,7]^. In severe chronic presentations where joint damage is extensive, salvage options including talocrural fusion have also been described^[7]^. Compared with these strategies, our approach combined open reduction with peroneus brevis tendon augmentation to address both chronic osseous malalignment and associated lateral ligament insufficiency. This technique offers several advantages: the peroneus brevis is a strong, local autograft harvested through the same incision, has low donor-site morbidity, and provides reliable biomechanical performance for ligament reconstruction^[6,7]^.

Postoperative care is vital for reconstructing functional ankle biomechanics. Rehabilitation protocols often incorporate early mobilization, progressive weight-bearing, and physiotherapeutic interventions aimed at restoring strength and enhancing functional capability while minimizing stiffness^[8]^. Regular follow-up assessments using imaging techniques may be warranted to monitor for complications such as hardware failure or malunion^[15]^.

## Conclusion

This case report highlights the rarity and diagnostic challenges of Bosworth fracture-dislocation, particularly in chronic or neglected cases. A novel surgical technique combining open reduction, syndesmotic screw fixation, and peroneus brevis tendon reconstruction effectively restored both bony stability and soft tissue function. This approach offers a successful management strategy for chronic Bosworth fracture-dislocation, leading to improved anatomical alignment and functional recovery.

## Data Availability

Supporting data will be available upon reasonable request.
